# Automatic diagnosis of melanoma using machine learning methods on a spectroscopic system

**DOI:** 10.1186/1471-2342-14-36

**Published:** 2014-10-13

**Authors:** Lin Li, Qizhi Zhang, Yihua Ding, Huabei Jiang, Bruce H Thiers, James Z Wang

**Affiliations:** 1 Department of Computer Science & Software Engineering, Seattle University, Seattle, WA 98122, USA; 2 Department of Biomedical Engineering, University of Florida, Gainesville, FL 32611, USA; 3 School of Computing, Clemson University, Clemson, SC 29634, USA; 4 Department of Dermatology, Medical University of South Carolina, Charleston, SC 29425, USA

## Abstract

**Background:**

Early and accurate diagnosis of melanoma, the deadliest type of skin cancer, has the potential to reduce morbidity and mortality rate. However, early diagnosis of melanoma is not trivial even for experienced dermatologists, as it needs sampling and laboratory tests which can be extremely complex and subjective. The accuracy of clinical diagnosis of melanoma is also an issue especially in distinguishing between melanoma and mole. To solve these problems, this paper presents an approach that makes non-subjective judgements based on quantitative measures for automatic diagnosis of melanoma.

**Methods:**

Our approach involves image acquisition, image processing, feature extraction, and classification. 187 images (19 malignant melanoma and 168 benign lesions) were collected in a clinic by a spectroscopic device that combines single-scattered, polarized light spectroscopy with multiple-scattered, un-polarized light spectroscopy. After noise reduction and image normalization, features were extracted based on statistical measurements (i.e. mean, standard deviation, mean absolute deviation, *L*_*1*_ norm, and *L*_*2*_ norm) of image pixel intensities to characterize the pattern of melanoma. Finally, these features were fed into certain classifiers to train learning models for classification.

**Results:**

We adopted three classifiers – artificial neural network, naïve bayes, and k-nearest neighbour to evaluate our approach separately. The naive bayes classifier achieved the best performance - 89% accuracy, 89% sensitivity and 89% specificity, which was integrated with our approach in a desktop application running on the spectroscopic system for diagnosis of melanoma.

**Conclusions:**

Our work has two strengths. (1) We have used single scattered polarized light spectroscopy and multiple scattered unpolarized light spectroscopy to decipher the multilayered characteristics of human skin. (2) Our approach does not need image segmentation, as we directly probe tiny spots in the lesion skin and the image scans do not involve background skin. The desktop application for automatic diagnosis of melanoma can help dermatologists get a non-subjective second opinion for their diagnosis decision.

## Background

Melanoma is a lethal form of skin cancer, with an estimated mortality rate of 14% worldwide [1]. The American Cancer Society reported 76,690 new cases of melanoma in the United States in 2013, with 9,480 estimated deaths, according to recent annual cancer facts and figures [2]. The global cancer statistics [3] also shows that the incidence and mortality rates of melanoma are in rising trend. Fortunately, melanoma may be treated successfully with a 10-year survival rate between 90 and 97% yet the curability depends on its early detection and excision when the tumor is still small and thin. Therefore, early and accurate diagnosis of melanoma is particularly important.

With the use of dermoscopy [4] and several clinical algorithms such as the ABCD rule [5], the 7-point checklist [6], and the Menzies method [7], the diagnosis accuracy of melanoma has been higher than the simple naked-eye examination [8]. However, clinical diagnosis is inherently subjective and complex, thus the accuracy is highly depending on experiences of dermatologists, which is estimated to be about 75 – 85% [9].

To reduce subjectivity and complexity of clinical diagnosis, it is desirable to conduct research on quantitative approaches for automated detection of melanoma. In the last two decades, a large amount of computer-aided approaches have been developed for diagnosis of melanoma. For example, A.G. Manousaki et al. [10] have proposed an approach that incorporates parameters of geometry, color and color texture as independent covariates for discriminating melanoma from melanocytic nevi. In the work of H. Ganster et al. [11], a melanoma recognition system that involves image processing, segmentation, feature calculation and selection, as well as k-NN classification has been presented. The work [12] by J.F. Alcón et al. also presents an automatic imaging system that combines the outcome of the image classification with context knowledge such as skin type, age, gender to add confidence to the classification. R. Garnavi and M. Aldeen have presented an approach [13] that uses border- and wavelet-based texture and four different classifiers in diagnosis of melanoma. Additionally, many computer applications have been developed for melanoma diagnosis. Some of these applications include SolarScan [14], the DANAOS expert system [15], DermoGenius-Ultra [16], and MelaFind [17], etc. These methods have achieved good classification accuracy, but either most of them obtained images by hand-held cameras, which thus needed image segmentation to separate the lesion from the background, or they were largely based on the light intensity spectra, derived absorption and scattering spectra, which have been shown to be highly sensitive to the abnormal changes in tissues. Moreover, most of these methods treat the skin tissue as a uniform or homogeneous medium.

Nevertheless, the skin tissue is inhomogeneous with multilayered structure. It consists of two primary layers that include a bottom layer – the dermis, and a top layer – the epidermis. Under the dermis is subcutaneous tissue, or hypodermis, which consists of connective tissue, fibroblasts, and fat cells. The epidermis consists of cells called keratinocytes, which develop in the basal layer of the skin at the bottom of the epidermis. As these keratinocytes migrate to the surface, they flatten, cornify, and harden, thusly sealing the skin [18]. The epidermis also includes melanocytes located near the base of the epidermis. These cells secrete the pigment melanin which protects the skin from ultraviolet radiation. The dermis can vary in thickness from 1 to 4 mm, and is primarily comprised of fibrous connective tissue such as collagen. The dermis also contains the hair follicles, sweat glands, blood vessels, and nerves. The skin is an extremely complex optical structure because it consists of multiple scatters with many shapes and sizes. As light propagates through the skin, it is scattered and absorbed differently in each layer. The absorption is also complex consisting of contributions primarily from blood and melanin. The multilayered structure further increases the complexity. Because skin cancer often begins in the epidermal layer and invades deeper tissue over time, the information obtained with the current multiple-scattered light-based methods is averaged out and does not reflect the accurate morphology of the specific diseased layer, although encouraging results for skin cancer detection have been recently obtained using multiple-scattered light-only methods in conjunction with classification algorithms [19,20].

Two techniques have been developed for use in deciphering the multilayered characteristics of human skin: multilayer model-based Multiple-Scattered Light Spectroscopy (MSLS) [21-25] and single-scattered, Polarized Light Spectroscopy (PLS) [26-31]. The multilayer model-based MSLS considers the realistic multilayered structure of the skin in the mathematical model of light propagation, thus is a good choice for skin studies. However, the accurate use of such improved spectroscopy requires knowing the exact thickness of each layer of skin which is difficult to obtain. On the other hand, PLS is able to physically discriminate between multiple layers of tissue, which tends to simplify the light propagation modeling because single-scattered light maintains its original polarization. Nevertheless, compared to MSLS, PLS has a relatively low signal-to-noise ratio.

To take the advantages of both MSLS and PLS, in this study, we captured skin images with a combination of MSLS and PLS technologies, and developed an automated method for diagnosis of melanoma via pattern classification of the skin images. The combined MSLS and PLS technologies provide unprecedented tissue functional information and cellular structures and accurately reflect morphologies in specific diseased layers of skin. Using a number of skin scans collected from a clinic by our spectroscopic system combining single and multiple-scattered light measurements, we first identified the pixel-by-pixel intensity differences between the group of melanoma and the group of benign skins. We then selected statistical measurements of pixel intensity that presented characteristics of melanoma as features for classification. Next, classification was carried out by using the selected features. We evaluated our approach using artificial neural network (ANN), naïve bayes (NB), and k-nearest neighbour (k-NN) separately. The approach could achieve 89% sensitivity, 89% specificity, and 89% accuracy by using NB. Based on the proposed method, a desktop application that runs on the spectroscopic system has been developed to integrate image acquisition, image processing, feature extraction, and classification into one-stop service. Dermatologists can use this application to conduct instantaneous diagnosis of melanoma to get a second opinion to their clinical decisions.

## Methods

**Figure 1:**
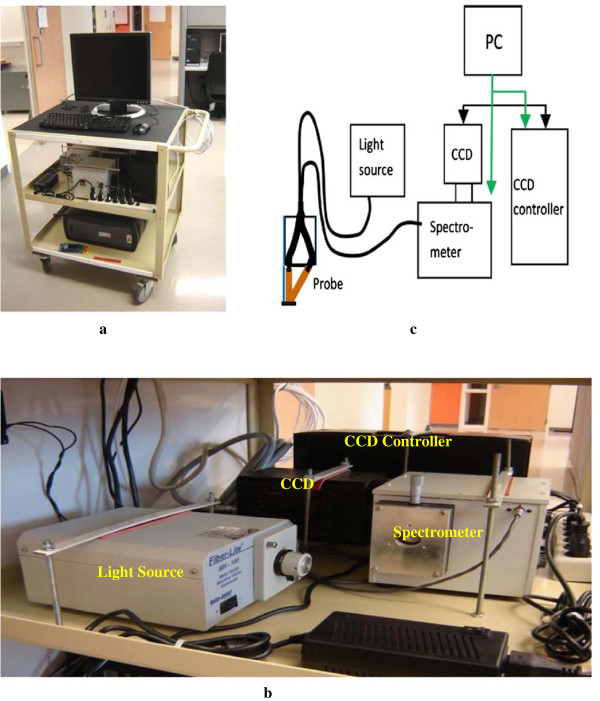
**Pictures of the spectroscopic system. (a)** front view of the system; **(b)** instruments on the 2nd layer of the cart; **(c)** system schema.

**Figure 2:**
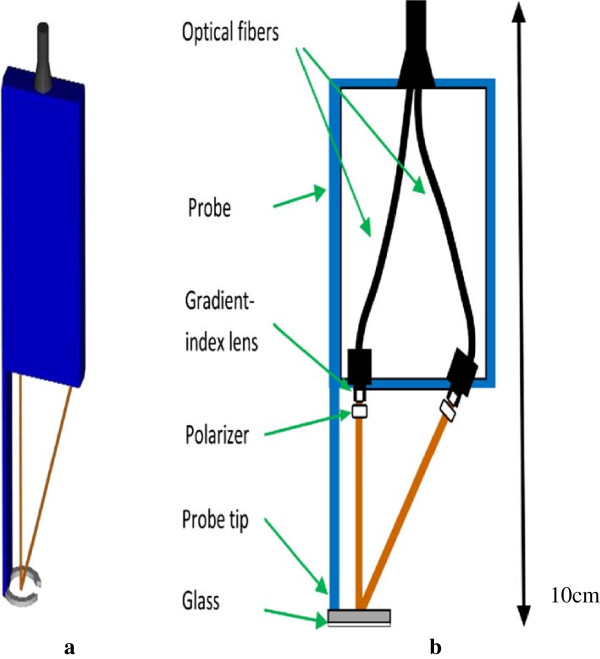
**A schema of the probe.** A three-dimensional view **(a)** and a two-dimensional schema **(b)** of the optical probe.

Data used in this work were collected in a clinic by a spectroscopic system with combined single and multiple-scattered light measurements. The study was approved by the Institutional Review Board (IRB) for Human Research in Medical University of South Carolina. Patients have consented to participate in the study and the study has not used patient identifiable information. The spectroscopic system was composed of a PC with a monitor, a CCD camera, a CCD controller, a spectrometer, a light source, optical fibers, a polarizer, gradient-index lens, and a probe. Figure 1 shows the pictures of the system, including the front panel removed to show the inside (1a), the layout of instruments inside the system (1b), and the system schema (1c). In the design of the optical probe, optical fibers with gradient-index lens built at the distal end were employed instead of attaching a separated lens system with the source and detector fibers. Figure 2 is a schema of the probe.

### Participants

The data set in this study included 187 samples from 79 participants, as some participants provided a couple of samples located in different areas of skin. The participants were aged from 22 to 79. The 187 samples consisted of 19 melanoma [16% female, 84% male, mean age = 61.32 (12.88) years] and 168 benign scans (i.e. benign nevus) [51% female, 49% male, mean age = 34.63 (11.40) years]. The data samples were sent for pathology and the labels (i.e. benign and melanomas) were confirmed correct.

### Image acquisition and preprocessing

**Figure 3:**
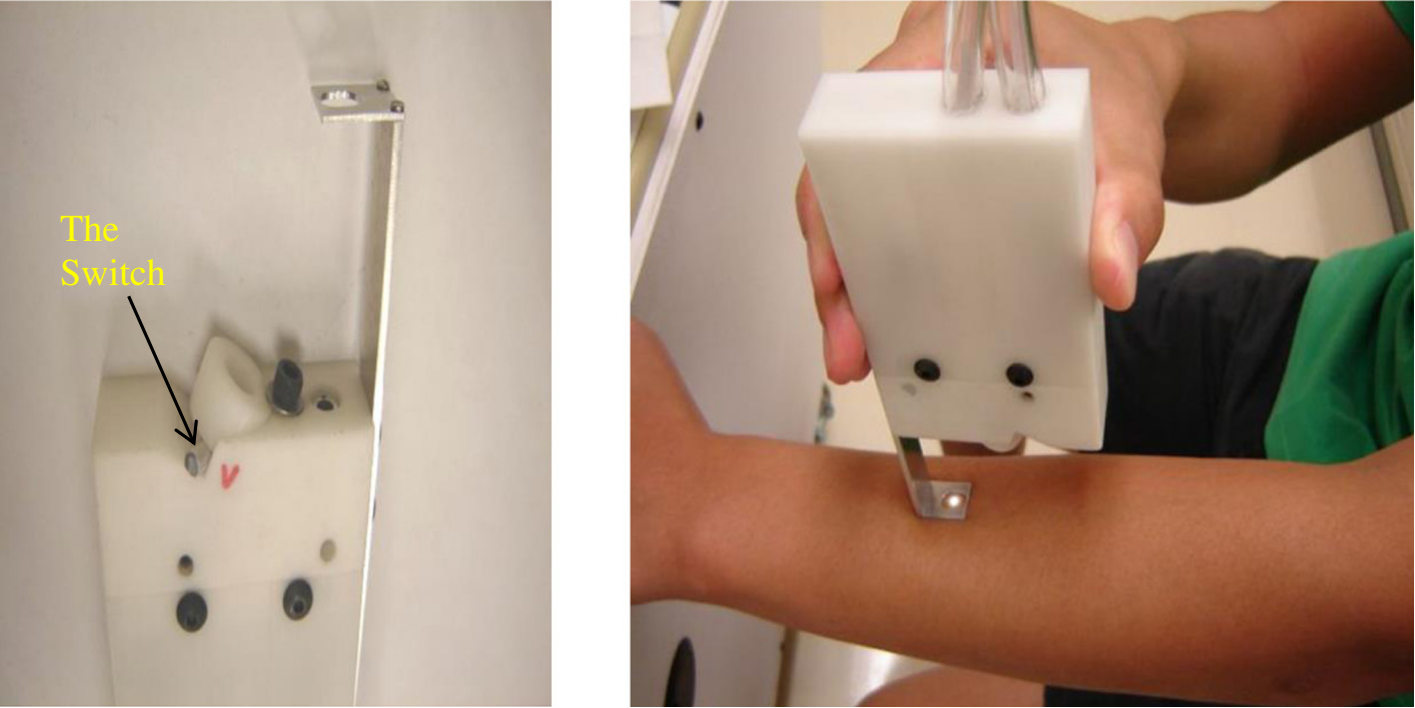
**Demonstration of image acquisition.** Left: the switch on the probe from which to perform either 'P’ scan or 'V’ scan. Right: we place the tip of the probe on the skin in a perpendicular way and let the light spot locate in the lesion area of the skin.

For each skin sample, our spectroscopic system gathered images from three spots. We performed two pairs of scans on each lesion skin area. Each pair included one 'P’ scanning (using parallel light) and one 'V’ scanning (using vertical light). If the lesion or abnormal area was larger than the probe tip, we moved the probe tip slightly to take the two pairs on two different spots of the lesion area. If the lesion area was too small, only one spot was chosen and scanned twice to obtain two pairs of scans. We also took one pair of scans on the normal skin area nearby for comparison. Therefore, six spectral images were collected for each sample. Figure 3 depicts the switch on the probe for selecting P scan or V scan (left) and the skin lesion imaging by a technician (right).

**Figure 4:**
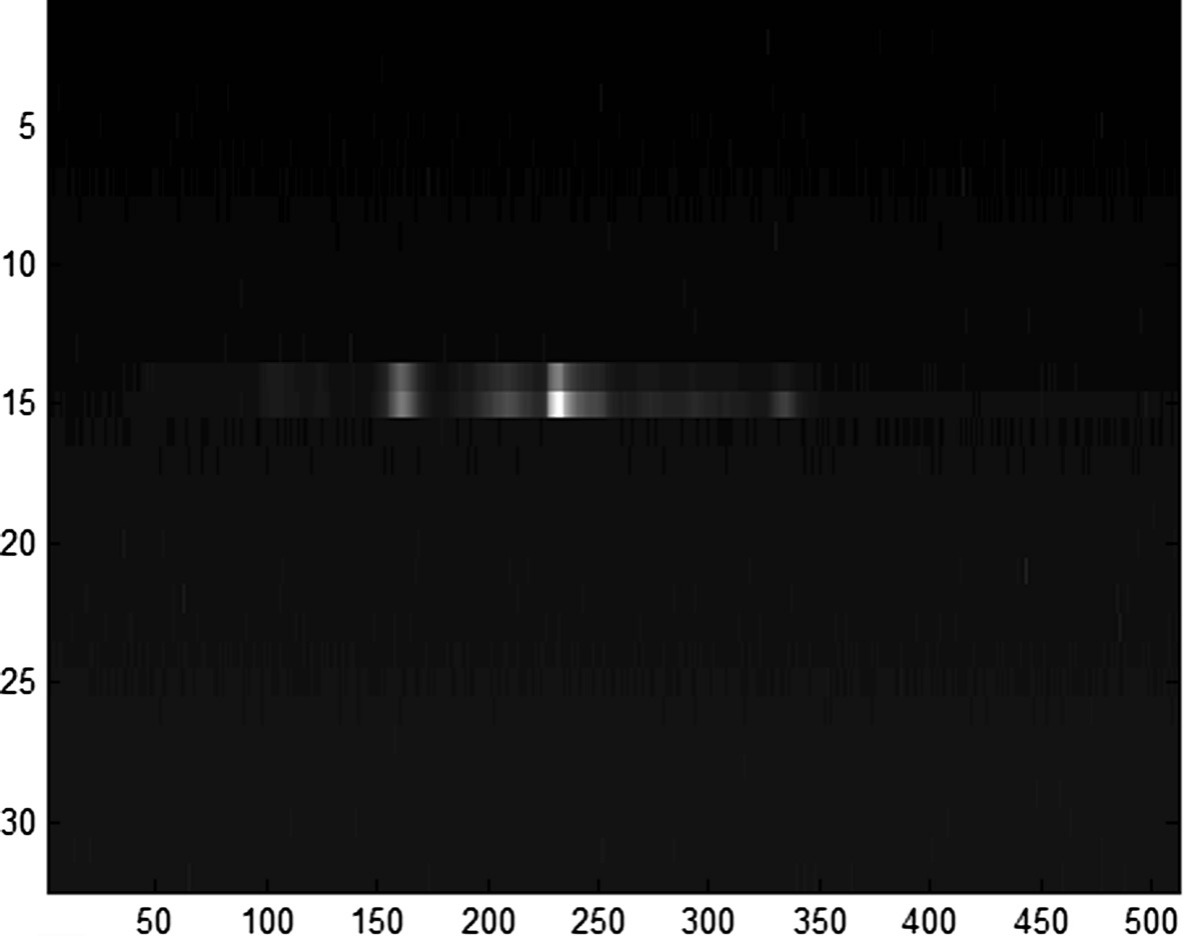
P scan of a melanoma skin lesion.

The spectral images are in a binary format. Each spectral image contains 32 × 512 pixels. The value of each pixel is the intensity obtained by the CCD camera. Considering that the images may contain noises due to environmental effects during collection, we performed noise reduction using the median filter. After noise reduction, pixel intensities were normalized with min-max normalization method, as shown in formula (1),

(1) $$I{'}_{\left(i,j,k\right)}=\frac{I_{\left(i,j,k\right)}-\min}{\max-\min}\left(\max'-\min'\right)+\min'$$

where *I*_(*i*,*j*,*k*)_ represents the original pixel intensity at position (i, j) of image k (1 ≤ i ≤32, 1 ≤ j ≤512 ), and *I* ' _(*i*,*j*,*k*)_ is the corresponding normalized intensity. *max* and *min* are the largest and smallest intensities in the original image respectively; *max* ' = *1* and *min* ' = *0*.Take P scan as an example, Figure 4 presents a P scan of a melanoma lesion, where we picked line 15 to visualize the spectra as pixels in this line have the most significant intensities. V scans also demonstrate similar spectra.

### Feature extraction

**Figure 5:**
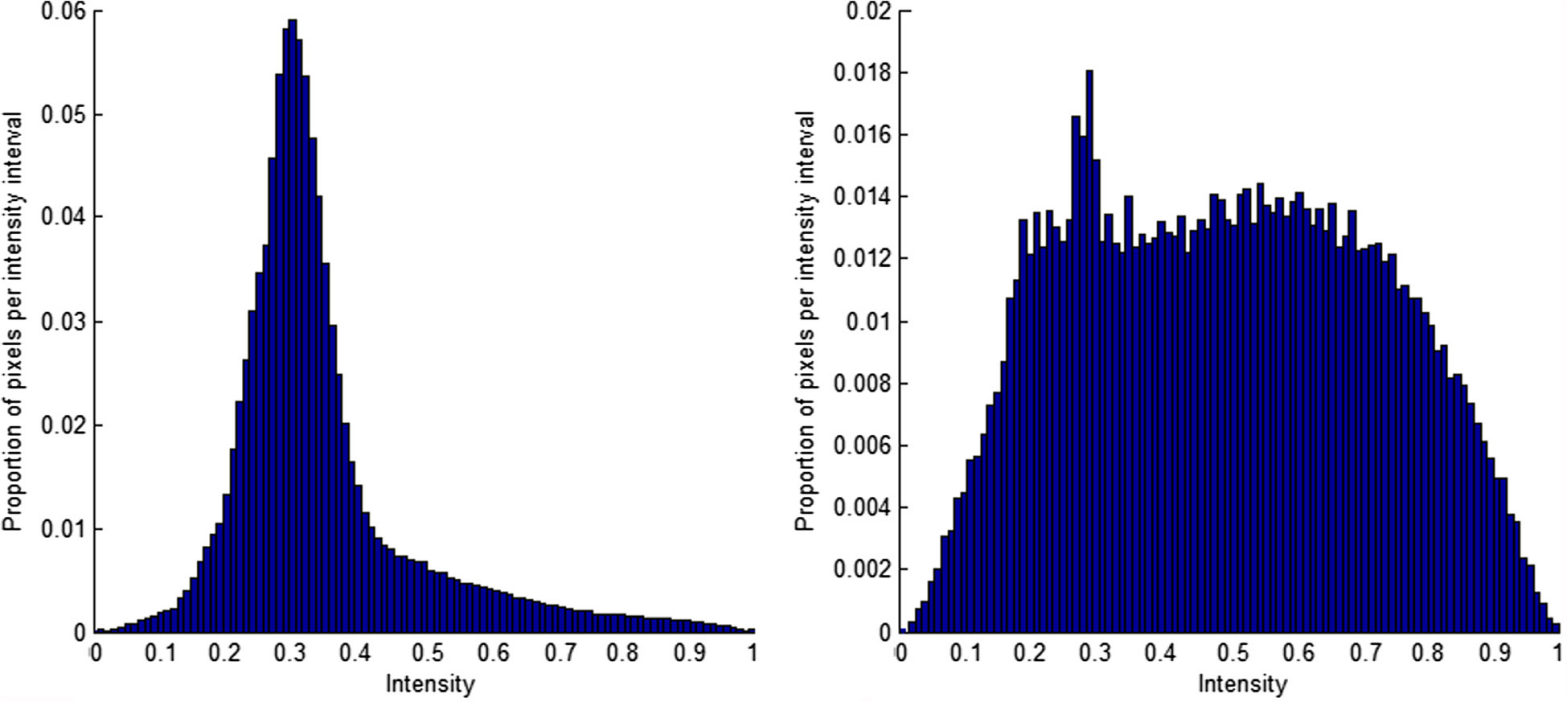
**Intensity distribution of P scans of all benign and all melanoma skin lesions.** Left: Intensity distribution of benign skin lesions; Right: intensity distribution of malignant melanoma skin lesions.

As the light generated by our spectroscopic system propagates through the benign and malignant skin, it is scattered and absorbed differently, which results to the intensity differences in the collected spectral images. Take P scan as an example, Figure 5 demonstrates the intensity distributions of all benign (left) and all malignant melanoma skin lesions (right). It is easy to find that the pixel intensities of malignant melanoma skin images spread out in the interval from 0 to 1, with most pixels having high intensity, whereas the majority of intensity values of benign skin images fall in the interval from 0.2 to 0.4. We also found that the intensity distribution of V scan has demonstrated similar differences between all benign and all melanoma skin lesions.

Since the intensity distributions of the benign and melanoma skin images are greatly different as shown in Figure 5, we therefore consider the pixel intensities as the key feature to distinguish benign skin from melanoma. To fully describe the pixel intensity distribution in each image, we adopted five statistical measures to quantify each scan. The five statistical variables are mean *μ*, standard deviation *σ*, mean absolute deviation MAD, *L*_1_ norm ${||{\overset{-}{I}}_{\left(i,j\right)}||}_{1}$, and *L*_2_ norm ${||{\overset{-}{I}}_{\left(i,j\right)}||}_{2}$, where ${\overset{-}{I}}_{\left(i,j\right)}$ is the corrected intensity of pixel (i, j). We calculated the statistical values separately for P scans and V scans. That is to say, each sample has 10 statistical measures (i.e. 5 for P scans and 5 for V scans). Additionally, as for the pixel intensity of P scans or V scans, in each sample, we took both 2 scans of lesion area and 1 scan of normal area nearby into consideration. The corrected intensity of pixel (i, j) in P scans of sample k, ${\overset{-}{I}}_{\left(i,j,k,p\right)}$, was computed by formula (2),

(2) $${\overset{-}{I}}_{\left(i,j,k,p\right)}\;=\;\left(I{'}_{\left(i,j,k,p1\right)}+I{'}_{\left(i,j,k,p2\right)}\right)/2\;-\;I{'}_{\left(i,j,k,p3\right)}$$

where *I* ' _(*i*,*j*,*k*,*p*1)_ is the normalized intensity of pixel (i, j) in p1 scan - P scan of the 1^st^ selected spot in the lesion area, *I* ' _(*i*,*j*,*k*,*p*2)_ is the normalized intensity of pixel (i, j) in p2 scan – P scan of the 2^nd^ selected spot in the lesion area, and *I* ' _(*i*,*j*,*k*,*p*3)_ is the normalized intensity of pixel (i, j) in p3 scan – P scan of a spot selected in the normal area nearby. The same computation was conducted on V scans. Measuring the pixel intensity with formula (2) can capture the difference of lesion skin and normal skin regarding the same subject, which makes more sense than using intensities of lesion skin alone, as intensity values may be influenced by factors like skin color, age, gender, etc.

We use the following formulas (3) – (7) to calculate the statistical values in P scans of sample k. In the formulas, m =32 and n =512 as there are 32 × 512 pixels per scan. The index i is in the range [1, 32], and the index j is in the range [1, 512]. Similarly, the formulas are applied to calculate the statistical measurements of V scans by replacing ${\overset{-}{I}}_{\left(i,j,k,p\right)}$ with ${\overset{-}{I}}_{\left(i,j,k,v\right)}$.

(3) $$\mu \;=\;\frac{1}{mn}\sum_{i=1}^{m}\sum_{j=1}^{n}{\overset{-}{I}}_{\left(i,j,k,p\right)}$$

(4) $$\sigma \;=\;\sqrt{\frac{1}{mn}\sum_{i=1}^{m}\sum_{j=1}^{n}{\left({\overset{-}{I}}_{\left(i,j,k,p\right)}-\mu \right)}^{2}}$$

(5) $$\mathrm{MAD}\;=\;\frac{1}{mn}\sum_{i=1}^{m}\sum_{j=1}^{n}\left|\;{\overset{-}{I}}_{\left(i,j,k,p\right)}-\mu \;\right|$$

(6) $${||{\overset{-}{I}}_{\left(i,j,k,p\right)}||}_{1}\;=\;\sum_{i=1}^{m}\sum_{j=1}^{n}\left|{\overset{-}{I}}_{\left(i,j,k,p\right)}\right|$$

(7) $${||{\overset{-}{I}}_{\left(i,j,k,p\right)}||}_{2}\;=\;\sqrt{\sum_{i=1}^{m}\sum_{j=1}^{n}{\left|{\overset{-}{I}}_{\left(i,j,k,p\right)}\right|}^{2}}$$

### Classification

After extracting the diagnostic features, we trained different classifiers (ANN, NB, and k-NN) to distinguish melanoma from benign skin. Given a training sample set with n samples $D={\left\{\left(x_{i},y_{i}\right)\right\}}_{i=1}^{n}$ where **x**_*i*_ is a sample and *y*_*i*_ is the associated class label, we focused on the binary classification problem, i.e., *y*_*i*_ is from a label space {±1} where +1 denotes the cancer class and -1 denotes the non-cancer class. The cancer class was composed of the samples of melanoma, whereas the non-cancer class consisted of the samples from benign skin. Each sample in the training set has 10 features to be fed into the classifier. The tool WEKA [32] was used for training and testing. With ANN, a multi-layer network that used back propagation was built. The input layer had 10 input units, which were the 10 selected features. The output layer had 2 units, representing two classes – benign and melanoma. The hidden layer was initially set to have 6 units in training as normally the number of hidden units is set to the half of the sum of input units (10 in our study) and output units (2 in our study). We kept the default parameters (i.e. learningRate = 0.3, momentum = 0.2, seed = 0, trainingTime = 500, validationThreshold = 20) in WEKA for ANN. As for NB, the classifier used estimator classes. Numeric estimator precision values were chosen based on analysis of the training data. The classifier used a normal distribution for numeric attributes. We kept the default parameters (i.e. useKernelEstimator = false, useSupervisedDiscretization = false) in WEKA for NB classifier. The choice of k in k-NN affects the performance of this classifier. In our study, we used 3-NN, which combines robustness to noise and costs less time for classification than using a larger k [33]. For other parameters of k-NN in WEKA, we set crossValidate to false, did not use distance weighting, and used the brute force search algorithm for nearest neighbour search.

## Results

### Pattern of melanoma

**Figure 6:**
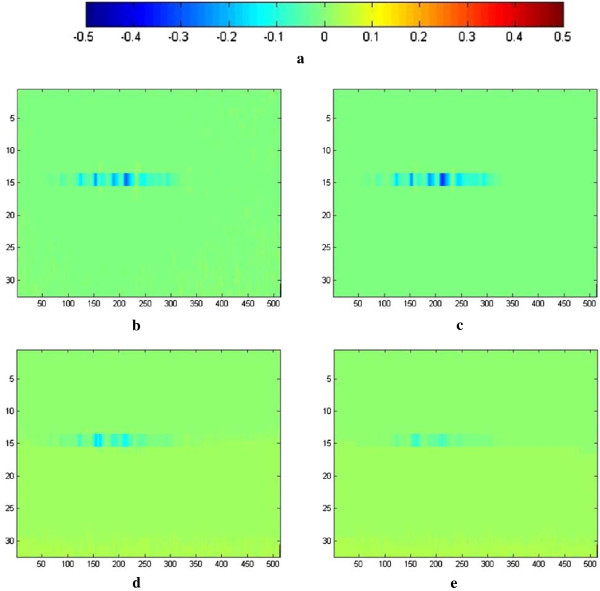
**Pattern of melanoma. (a)** The color bar representing the color scheme used for pixel intensity; **(b)** The color map of pixel intensity calculated by formula (2) in P scan of a benign case; **(c)** The color map of pixel intensity calculated by formula (2) in V scan of the same benign case as that was used in **(b)**; **(d)** The color map of pixel intensity computed by formula (2) in P scan of a melanoma case; **(e)** The color map of pixel intensity computed by formula (2) in V scan of the same melanoma case as that was used in **(d)**.

Melanoma and benign group comparison across the 10 features demonstrated the characteristic of melanoma. The effect of melanoma can be seen in the distribution of the pixel intensity differences of abnormal skin and normal skin of individual subjects. Figure 6 demonstrates that the influence on melanoma in terms of pixel intensity can be observed in color scale by comparing a melanoma case to a benign case.

### Classification accuracy

In our experiment, the 187 cases were randomly divided into a training set of 60 cases and a test set of 127 cases by random sampling without replacement. We conducted the experiment using three classifiers (i.e. ANN, NB, and 3-NN) separately. The experiment was repeated 25 times for each classifier. In addition, we evaluated the performance of classification using P scan alone (i.e. 5 statistical measures of P scan for classification), V scan alone (5 statistical measures of V scan for classification), as well as the combined P scan and V scan (10 features for classification). Table 1 demonstrates the performance of using NB with the combined P scan and V scan, of which, on average, the accuracy, specificity, and sensitivity were 89%, 89%, 89% respectively. The probability of error in NB was 0.16. The average accuracy, specificity and sensitivity of using ANN with the combined P scan and V scan were 88%, 93%, and 49% respectively. 3-NN with the combined P scan and V scan demonstrated 88% accuracy, 92% specificity, and 47% sensitivity on average. The probability of error in ANN and 3-NN was 0.24 and 0.26 respectively. Table 2 shows the average performance of the classifiers using P scan and V scan, using P scan alone or using V scan alone. Due to space limitation, the performance details of each run of experiment with different classifiers (i.e. ANN, 3-NN) and different sets of features (i.e. P scan alone or V scan alone) were skipped. In addition, we performed the experiment with a different combination of data by randomly dividing the data into a training set of 30 cases and a test set of 157 cases. The average performance of each classifier using P scan and V scan, using P scan alone or using V scan alone is shown in Table 3.

**Table 1:** Performance of NB using P scan and V scan together (60 training samples, 127 testing samples)

	**Sensitivity**	**Specificity**	**Accuracy**
Run #1	100	86	87.4
Run #2	76.9	92.1	90.6
Run #3	100	91.2	92.1
Run #4	84.6	91.2	90.6
Run #5	76.9	88.6	87.4
Run #6	92.3	87.7	88.2
Run #7	100	87.7	89
Run #8	100	88.6	89.8
Run #9	100	87.7	89
Run #10	100	89.5	90.6
Run #11	69.2	89.5	87.4
Run #12	76.9	90.4	89
Run #13	92.3	89.5	89.8
Run #14	92.3	90.4	90.6
Run #15	76.9	88.6	87.4
Run #16	76.9	94.7	92.9
Run #17	84.6	89.5	89
Run #18	100	87.7	89
Run #19	92.3	87.7	88.2
Run #20	84.6	87.7	87.4
Run #21	92.3	90.4	90.6
Run #22	100	91.2	92.1
Run #23	100	85.1	86.6
Run #24	100	86.8	88.2
Run #25	46.2	93	88.2
Average	88.6	89.3	89.2

All values are in %.

**Table 2:** The average performance of the classifiers using P scan and V scan, using P scan alone or using V scan alone (60 training samples, 127 testing samples)

	**Sensitivity**	**Specificity**	**Accuracy**
NB (using P & V scan)	88.6	89.3	89.2
NB (using P scan)	83.7	77.8	78.4
NB (using V scan)	95.7	85.1	86.2
ANN (using P & V scan)	49.2	92.8	88.4
ANN (using P scan)	13.2	96.6	88.2
ANN (using V scan)	19.7	96.9	89.0
3NN (using P & V scan)	46.8	92.1	87.5
3NN (using P scan)	24.6	93.3	86.3
3NN (using V scan)	32.0	95.6	89.0

All values are in %.

**Table 3:** The average performance of the classifiers using P scan and V scan, using P scan alone or using V scan alone (30 training samples, 157 testing samples)

	**Sensitivity**	**Specificity**	**Accuracy**
NB (using P & V scan)	73.0	90.8	89.0
NB (using P scan)	74.0	82.2	81.4
NB (using V scan)	81.8	86.0	85.6
ANN (using P & V scan)	66.5	89.8	87.4
ANN (using P scan)	30.0	94.2	87.8
ANN (using V scan)	49.0	92.8	88.3
3NN (using P & V scan)	70.3	89.7	87.7
3NN (using P scan)	28.8	92.6	86.1
3NN (using V scan)	51.0	92.3	88.1

All values are in %.

### Desktop application

**Figure 7:**
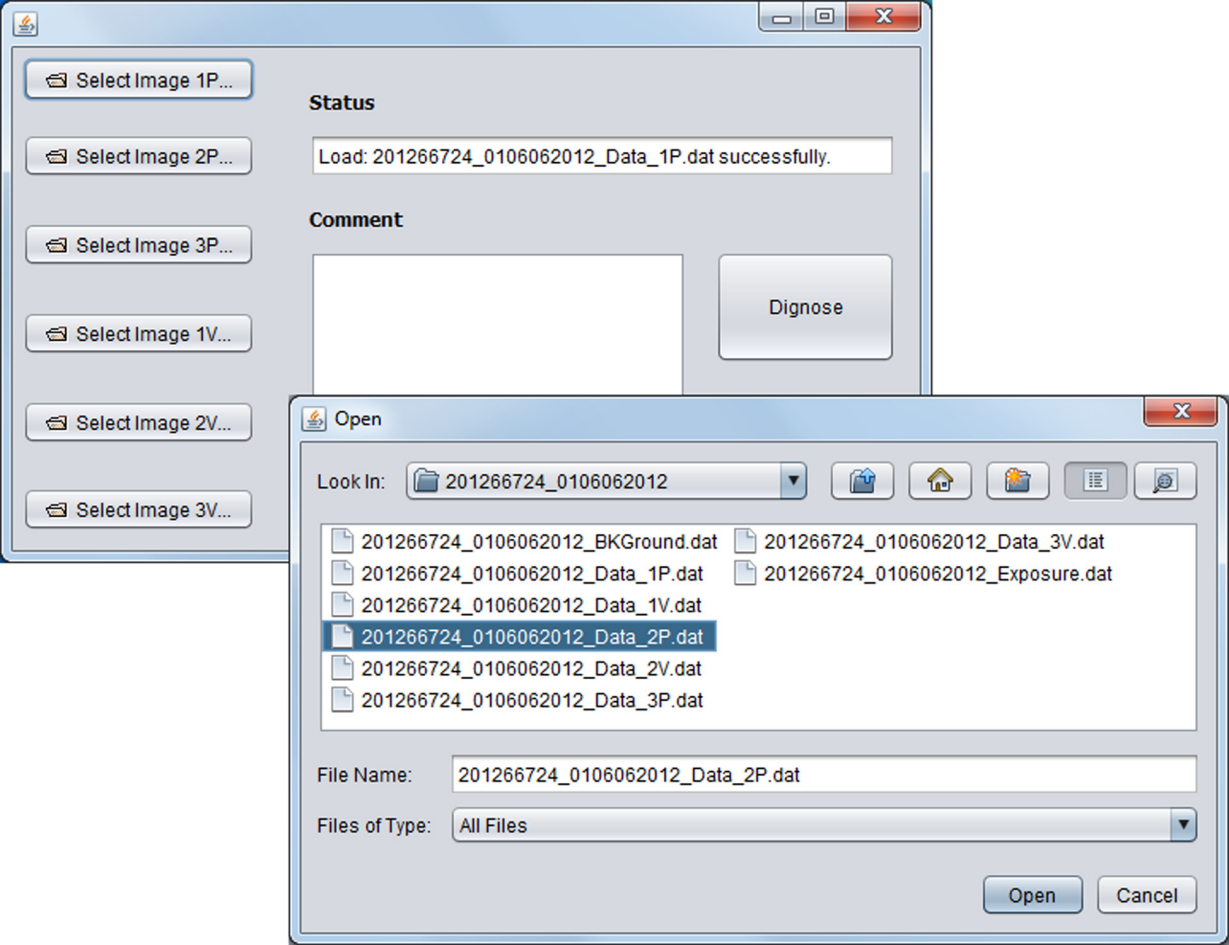
A screenshot of the desktop application for automated diagnosis of melanoma.

For automatic detection of melanoma, we built a desktop application which runs on the machine (Figure 1a) that connects to the spectroscopic device. Clinicians can launch the application to collect skin scans using the spectroscopic device, and follow the wizard to make the application automatically conduct image processing, feature extraction, and classification so as to achieve instantaneous diagnosis of melanoma. The NB classifier was integrated in this application. Figure 7 presents a screenshot of the automated tool. We keep the interface simple for dermatologists to use. Dermatologists select images 1P, 2P, 3P and 1 V, 2 V, 3 V that have been collected by the spectroscopic device for a subject, by clicking on the 6 buttons on the very left panel of the interface. The status box shows whether an image is loaded successfully. Users can input and save comments for the diagnosis in the “Comment” area. Once images are all loaded successfully, users just need to click on the “Diagnose” button to start the diagnosis, which launches the execution of back-end programs for feature extraction and classification. Once the diagnosis is done, a message box pops up showing the diagnosis result – either melanoma or benign.

## Discussion

Computer-aided diagnosis of melanoma generally involves six steps: image acquisition, image processing, image segmentation, feature extraction, feature selection, and classification. In our work, we gathered images with combined single and multiple-scattered light measurements, for deciphering the multilayered characteristics of human skin. This is one scientific contribution of our work. The other strength of our work is that scans of lesion area do not involve background skin as we directly probe tiny spots in the lesion skin. Image segmentation was skipped in our work. The objective of segmentation in diagnosis of melanoma is to detect the border of the lesion so as to separate the lesion – region of interest from the background skin. Since we use the probe only within the region of interest (ROI), our approach does not need image segmentation. An approach without segmentation reduces the risk of diagnosis error that may be introduced by improper segmentation and reduces the cost and time caused by segmentation. In addition, we did not perform feature selection because the 10 statistical parameters extracted for each sample were all needed for classification.

Classification methods that have been applied to computer-aided diagnosis of melanoma include discriminant analysis [34,35], ANN [36,37], k-NN [11], support vector machine (SVM) [38], and decision trees, etc. To evaluate our approach, we performed classification using three different classifiers: ANN, NB and k-NN, with the combination of P scan and V scan, P scan alone, or V scan alone. Compared with ANN and k-NN, NB could achieve the best accuracy and sensitivity. Although ANN and k-NN achieved better specificity than NB, the sensitivity was not good. With NB, the experiment using the combination of P scan and V scan achieved better accuracy and specificity than using P scan or V scan alone, while the experiment with V scan alone presented the best sensitivity. We picked the classifier NB using the combined P scan and V scan which achieved the highest accuracy and integrated it in our application to provide automated diagnosis of melanoma.

There are some limitations in our work. First, although our approach could achieve 89% sensitivity, 89% specificity, and 89% accuracy with NB, the sensitivity with ANN and k-NN were not good enough. The small number of melanomas and much larger set of benign samples might make the benign samples dominate the classification. In addition, because of the limited number of melanomas in our experiment, it is likely that the performance of our approach is specific to the data sets used in this preliminary study. It is unclear how the performance varies when our approach is applied to a larger or/and new data set. Follow-up studies that incorporate larger size of melanoma may have the most reliability and provide greater confidence for the diagnosis than the classifier developed in this preliminary study. Second, some factors like the changes in the device, the person who uses the device, etc. might have effect on the outcome. Particularly, variation in skin color, age, gender in the sample set may influence the diagnosis result.

## Conclusion

This paper presents a computer-aided approach for automatic and accurate diagnosis of melanoma. We evaluated our approach with three classifiers - ANN, NB, and k-NN, and our approach could achieve 89% sensitivity, 89% specificity, and 89% accuracy with NB. In the future, we will do follow-up study by collecting more real data, especially melanoma cases, to further evaluate our approach.

## Abbreviations

ANN: Artificial neural network; K-NN: K-Nearest neighbour; MAD: Mean absolute deviation; MSLS: Multiple-scattered light spectroscopy; NB: Naïve bayes; PLS: Polarized light spectroscopy; SVM: Support vector machine.

## Competing interests

The authors declare that they have no competing interests.

## Authors’ contributions

LL implemented the image acquisition, performed image processing, designed the approach for feature extraction and classification, carried out the evaluation, developed the desktop application, and drafted the manuscript. QZZ developed the spectroscopic device. YHD conducted the extraction of features. HBJ designed the spectroscopic approach for image acquisition. BHT collected real data set in this study. JZW led the design of the study. All authors read and approved the final manuscript.

## Pre-publication history

The pre-publication history for this paper can be accessed here:

http://www.biomedcentral.com/1471-2342/14/36/prepub
